# Pharmacokinetic and Bioavailability Studies of Galgravin after Oral and Intravenous Administration to Rats Using HPLC-MS/MS Method

**DOI:** 10.1155/2021/9919789

**Published:** 2021-07-24

**Authors:** Lulu Zhao, Songrui Wang, Xuhua Huang, Yuqi Fan, Zixiang Xue, Dongyue Yang, Huizi Ouyang, Yanxu Chang, Jun He

**Affiliations:** ^1^First Teaching Hospital of Tianjin University of Traditional Chinese Medicine, 300193 Tianjin, China; ^2^National Clinical Research Center for Chinese Medicine Acupuncture and Moxibustion, 300193 Tianjin, China; ^3^State Key Laboratory of Component-Based Chinese Medicine, Tianjin University of Traditional Chinese Medicine, Tianjin 301617, China

## Abstract

This paper presents a new high-performance liquid chromatography tandem mass spectrometry (HPLC-MS/MS) method with a rapid analysis of 6 min to determine the concentration of galgravin in rat plasma so as to study its pharmacokinetic features and bioavailability in vivo. Schisandrin was selected as the internal standard (IS). After extracting the analyte from plasma samples with ethyl acetate, methanol-H_2_O (0.1% formic acid) (85 : 15, *v*/*v*) was used as mobile phase to achieve chromatographic separation on a C18 reversed phase column. The MS detection was performed in positive ion mode using electrospray ionization (ESI) source. This method showed good linearity over the range of 1~500 ng/mL (*R*^2^ > 0.999), and the lower limit of quantitation (LLOQ) was 1.0 ng/mL. The intraday precision and interday precision were both within 8.5%, whereas the accuracies were in the range of -2.6%–6.0%. The average recoveries of galgravin in rat plasma were between 92.3% and 99.3%. Moreover, galgravin was stable throughout storage and processing with all RSDs below 12.1%. After the successful application of this optimized method, the oral bioavailability of galgravin was determined to be 8.5%. This study will be helpful to the future research and development of galgravin.

## 1. Introduction

Lignans have attracted extensive attention of researchers owing to their wide bioactivities including antioxidant, antitumor, and hepatoprotective effects, as well as antileishmanial and antimalarial activities in recent years [1–4]. Galgravin is a new tetrahydrofuran lignan, which has been found in some medicinal plants, such as *Piper wallichii* [5], leaves of *Tridax procumbens* [6], rhizomes of *Acorus tatarinowii* Schott [7], and the stems of *Schisandra propinqua* [8]. Modern pharmacological studies have found that galgravin can effectively inhibit platelet activating factor and possesses anti-inflammatory and analgesic activities [9, 10]. In addition, galgravin showed significant cardioprotective and neuroprotective activities [11, 12]. Galgravin also inhibits excessive bone resorption by inhibiting differentiation of osteoclast, which plays a significant role in bone protection [13]. Evidence suggests that the compound displays promising cytotoxic activity against HL-60 cells (human leukemia) in vitro and might become a potential anticancer drug [14].

Currently, there are few literature reports on galgravin. In addition to using ^1^H and ^13^C NMR to identify the structure of this compound, the researchers once used vacuum liquid chromatography, semipreparative HPLC system [10], and supercritical fluid chromatography [15] during the extraction and isolation of the active ingredients in which galgravin was included. The GC (gas chromatography) and HPLC were also applied to determine the content of galgravin in the effective part of Chinese herbs [11, 16].

Although galgravin has a variety of pharmacological activities, there is no literature on its pharmacokinetics in vivo. It is widely known that HPLC-MS/MS method is much useful in the pharmacokinetic study of quantifying the main components of drugs because of its high selectivity and sensitivity. A new and efficient HPLC-MS/MS analysis method was established in this research and first successfully applied to investigate the pharmacokinetics and bioavailability of galgravin in two different ways of administration (intragastric and intravenous dosing). The study would provide some references for further research and application of this compound.

## 2. Materials and Methods

### 2.1. Reagents and Chemicals

**Figure 1:**
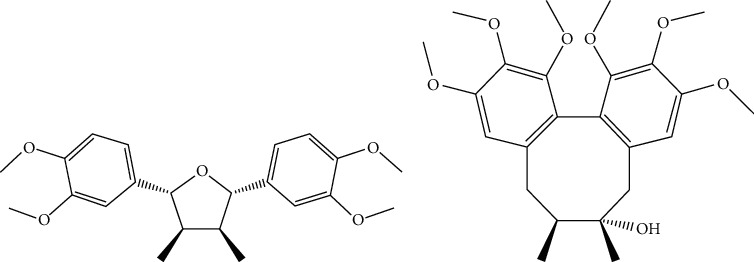
The chemical structures of (a) galgravin and (b) IS.

Chromatographic grade methanol and acetonitrile were obtained from Thermo Fisher Scientific Co. Ltd. (Canada). Both ethyl acetate (Concord Technologies Ltd., Tianjin, China) and formic acid (ROE Co. Ltd., Newark, USA) were also of HPLC grade. Ultrapure water was prepared by a Milli-Q water purification system (Millipore, Milford, MA, USA). Galgravin (purity ≥ 98%) and schisandrin (purity ≥ 99%) were purchased from Chengdu Dexter Biotechnology Co. Ltd. The chemical structures of galgravin and IS are presented in Figure 1.

### 2.2. Chromatographic and Mass Spectrometry Conditions

The separation and detection of the analyte were carried out using the LC-MS/MS system, which comprises mainly of an Agilent-1200 high-performance liquid chromatography system (Agilent, USA) and an Agilent-6430 triple quadrupole tandem mass spectrometer (Agilent, USA).

The chromatographic separation was achieved by using a Waters XBridge™ BEH C18 column (2.5 *μ*m, 4.6 × 50 mm) with column temperature of 25°C and a run time of 6 min. The mobile phase system was composed of methanol and 0.1% formic acid water (85 : 15, *v*/*v*). 5 *μ*L sample was injected into HPLC-MS/MS system under the flow rate of 0.3 mL/min.

The positive ESI with multiple reaction monitoring (MRM) mode was applied for mass detection. The specific parameters of galgravin and IS are shown in Table 1, and other parameters were optimized and set as follows: atomizer pressure was 25 psi, capillary temperature was 320°C, capillary voltage was 4000 V, and flow rate of drying gas was 11 L/min.

### 2.3. Calibration Standard (CS) and Quality Control (QC) Sample Preparation

A certain amount of galgravin and IS reference material were precisely weighed and dissolved with methanol to prepare respective stock solution at concentration of 1 mg/mL. Then, the stock solution of galgravin was diluted serially with methanol to prepare the working solutions over the concentration range of 5-2500 ng/mL. And IS working solution was at 200 ng/mL. All solutions were stored in a refrigerator at -20°C. Appropriate amount of working solution was added into blank rat plasma (100 *μ*L) for preparation of serial CS solutions, yielding final concentrations of 1, 2, 5, 10, 25, 50, 100, 250, and 500 ng/mL for galgravin and 40 ng/mL for IS. The concentrations of QC samples for method validation were 2, 50, and 400 ng/mL, respectively.

### 2.4. Plasma Sample Preparation

After addition of 100 *μ*L rat plasma, 20 *μ*L methanol, and 20 *μ*L IS working solution, the mixture was vortexed for 40 s; 1 mL ethyl acetate was then added into the centrifuge tube. It took 10 minutes to centrifugate the mixture under the condition of 12,000 × *g* and 10°C after vortex mixing of 3 min. The supernatant fraction was then transferred into another tube and evaporated to dryness under a gentle nitrogen stream. The obtained residue was redissolved with 100 *μ*L methanol, followed by vortex for 3 min and centrifugation for 10 min at 12,000 × *g*. Lastly, 5 *μ*L of the supernatant fluid was used to analyze with HPLC-MS/MS.

### 2.5. Method Validation

#### 2.5.1. Specificity

The specificity of method was investigated by comparing chromatograms of blank plasma samples from 6 rats with blank plasma samples spiked with galgravin and IS and an experimental plasma sample from a rat at 1 h after gavage of galgravin.

#### 2.5.2. Linearity and Sensitivity

The calibration curve was constructed at nine concentration levels (1-500 ng/mL for galgravin), and regression equation was calculated with the concentration of galgravin as independent variable (*x*), the peak area ratio of galgravin to IS as dependent variable (*y*), and 1/*x* as weight factor. Then, the correlation coefficient (*R*^2^) was used to evaluate the linearity. The sensitivity of the method was reflected by LLOQ with signal-to-noise ratio (SNR) greater than 10. At this concentration, not only should the relative error (RE) be within ±20% but also the relative standard deviation (RSD) was less than 20%.

#### 2.5.3. Precision and Accuracy

QC samples at three concentrations (*n* = 6) were prepared and analyzed continuously for 3 days to investigate intra- and interday precision and accuracy. The precision and accuracy are expressed by RSD and RE values, respectively. Meanwhile, the acceptance criteria were RSD ≤ 15% and RE within ±15%.

#### 2.5.4. Extraction Recovery and Matrix Effect

The peak area ratio of the galgravin-spiked blank plasma sample to the analyte added in postextracted blank matrix was used to investigate the extraction recovery, and the peak area ratio of the postextraction matrix spiked with the analyte to the nonextracted QC standard solution injected directly was used to study the matrix effect.

#### 2.5.5. Stability

In this study, the investigated stability items of QC samples (2, 50, and 400 ng/mL) were as follows: short-term stability (2 h at room temperature), freeze-thaw stability (three freeze-thaw cycles), postpreparative stability (12 h in autosampler), and long-term stability (1 week at -80°C).

### 2.6. Experimental Study on Pharmacokinetics

Twelve male Sprague-Dawley rats (250 ± 10 g, SPF grade) were randomly divided into two groups and fed in suitable environment (23 ± 3°C, relative humidity 50 ± 10%). After fasting overnight, the first group of rats received a single gavage administration of galgravin suspension (0.5% CMC-Na as solvent) at 20 mg/kg, and serial blood samples (about 250 *μ*L) were obtained from the orbital fossa vein into heparinized centrifuge tubes at prodose, 0.03, 0.08, 0.17, 0.5, 0.75, 1, 2, 4, 6, 8, 10, 12, 24, and 36 h after administration. The other group was injected with galgravin solution via tail vein. The dosage was 2 mg/kg, and blood samples were collected at prodose, 0.03, 0.08, 0.17, 0.5, 0.75, 1, 2, 4, 6, 8, 10, and 12 h. After centrifugation at 3,000 × *g* for 10 min, the plasma samples were immediately separated, followed by transfer into clean tubes and storage at −80°C. The animal studies were approved by the Animal Ethics Committee of Tianjin University Traditional Chinese Medicine (TCM-LAEC20200046).

### 2.7. Data Analysis

The plasma concentration of galgravin was quantitatively calculated using MassHunter Workstation software (version B.09.00). DAS Software (DAS 3.0; Medical College of Wannan, China) was applied to evaluate the exact pharmacokinetic parameters. The area under the concentration-time curve (AUC) is essential for the calculation of oral absolute bioavailability (*F*, %) using the following formula: *F* (%) = (AUC_i.g._ × Dose_i.v._)/(AUC_i.v._ × Dose_i.g._) × 100.

## 3. Results and Discussion

### 3.1. Optimization of LC-MS/MS Conditions

In this study, CORTECS™ C18 column (2.7 *μ*m, 2.1 × 50 mm), ZORBAX Eclipse C18 column (3.5 *μ*m, 2.1 × 100 mm), and X Bridge™ BEH C18 column (2.5 *μ*m, 4.6 × 50 mm) were investigated during the method development. The results showed that the peak shape of the analyte obtained by CORTECS™ C18 column (2.7 *μ*m, 2.1 × 50 mm) was asymmetric compared to X Bridge™ BEH C18 column (2.5 *μ*m, 4.6 × 50 mm), and the chromatographic response significantly reduced using ZORBAX Eclipse C18 column (3.5 *μ*m, 2.1 × 100 mm) with longer analysis time compared to X Bridge™ BEH C18 column (2.5 *μ*m, 4.6 × 50 mm). Therefore, the X Bridge™ BEH C18 column (2.5 *μ*m, 4.6 × 50 mm) was selected finally. Meanwhile, the influence of different mobile phase combinations (methanol-water, acetonitrile-water) or added buffers (phosphoric acid, formic acid) on the chromatographic behavior of the target compound was investigated. Methanol and 0.1% formic acid water were selected because of the symmetric peak shape, appropriate retention time, and detection sensitivity. The optimal column temperature was 25°C. The major MS spectrometry parameters were optimized individually to obtain better responses. Results showed that the signal response of galgravin and IS in positive ion mode was higher in contrast to the negative mode. In addition, the optimal ion transition pairs for quantitation were at m/z 373.3 ⟶ 235.2 for galgravin and m/z 433.2 ⟶ 384.3 for IS in MRM mode.

### 3.2. Investigation of Sample Preparation Method

Three extraction methods were investigated to find an appropriate preparation method of plasma sample in this study. It was found that liquid-liquid extraction (LLE) with ethyl acetate provided better recovery and process efficiency of galgravin compared to protein precipitation with methanol and acetonitrile. Hence, LLE was selected to extract the analyte from biological samples.

### 3.3. Validation of Analytical Method

#### 3.3.1. Specificity

**Figure 2:**
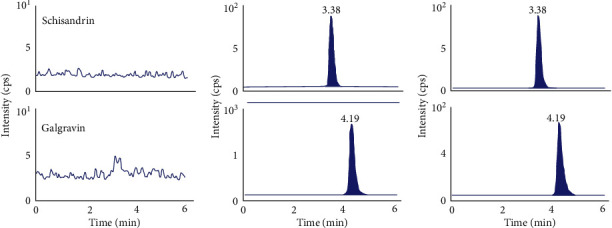
The typical MRM chromatograms of galgravin and IS: (a) blank plasma; (b) blank plasma spiked with galgravin and IS; (c) a rat plasma sample after oral administration of galgravin.

The typical MRM spectra of blank plasma (a), blank plasma spiked with galgravin and IS (b), and plasma sample after gavage (c) are displayed in Figure 2. There was not obvious endogenous plasma matrix interference found at the retention time of galgravin (4.19 min) and IS (3.38 min).

#### 3.3.2. Linearity and Sensitivity

Good linearity was attained in the range of 1-500 ng/mL. The typical regression equation of calibration curve was *y* = 0.020756*x* + 0.005814 (*R*^2^ > 0.999) by weighted least squares linear regression. And the LLOQ was established as low as 1.0 ng/mL, which indicated that the method has high sensitivity.

#### 3.3.3. Precision and Accuracy

The results of intra- and interday precision and accuracy are shown in Table 2. All RSD values of QC samples did not exceed 8.5% and RE ranged from -2.6% to 6.0%. The results were within acceptable limits of precision and accuracy.

#### 3.3.4. Extraction Recovery and Matrix Effect

As listed in Table 3, the mean extraction recovery of galgravin was 94.4%, 99.3%, and 92.3% at 2, 50, and 400 ng/mL, respectively. No significant signal suppression or enhancement was observed with this method. The data of matrix effect ranged from 83.1 ± 5.1% to 100.0 ± 6.1%.

#### 3.3.5. Stability

Table 4 sums the stability data of QC samples under different conditions. The results suggested that the analyte has good stability with all RSDs ≤ 12.1%.

### 3.4. Application of Analytical Method

**Figure 3:**
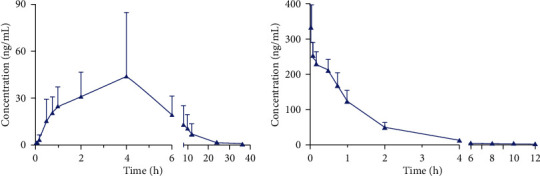
Mean plasma concentration-time profiles following (a) oral and (b) intravenous administration of galgravin to SD rats (mean ± SD, *n* = 6).

The novel analytical method was proved to be successful to determine the plasma samples obtained from rats that received intragastric and intravenous administrations of galgravin.  Figure 3 illustrates the trend of plasma concentration with time. The major pharmacokinetic parameters were calculated according to noncompartment model and presented in Table 5 in the form of mean ± standard deviation (SD).

The concentration of galgravin in plasma reached its peak (*C*_max_, 48.42 ± 37.66 ng/mL) at 2.08 h (*T*_max_) and declined with the *T*_1/2_ of 3.99 h after a single oral administration to rats (20 mg/kg), suggesting that galgravin had a moderate speed of absorption and metabolism in blood circulatory system after oral administration. However, *C*_max,oral_ was much lower than *C*_max,i.v._, which means that the absorption of galgravin was not very well. The AUC_(0 − *t*)_ (314.25 ± 179.84 h ng/mL) was close to the AUC_(0 − ∞)_ (315.75 ± 179.70 h ng/mL), which indicated that the monitoring time of this study was reasonable. After intravenous injection of galgravin solution at 2 mg/kg, the *C*_max_ of 332.80 ± 63.59 ng/mL was achieved at 0.03 h without absorption process; then, plasma concentration of galgravin decreased exponentially. Short MRT (1.27 ± 0.19 h) and *T*_1/2_ (1.34 ± 0.21 h) suggested fast elimination tendency of galgravin. The AUC_(0 − *t*)_ and AUC_(0 − ∞)_ were 369.56 ± 66.06 h ng/mL and 371.36 ± 66.59 h ng/mL, respectively. Finally, the calculated oral bioavailability of galgravin was 8.5%.

Based on the above results, the pharmacokinetic characteristics of galgravin may affect its clinical application in the future. Its insufficient oral absorption could be compensated by increasing the administered dose, and the longer retention time in the body may keep the drug in effect for a longer period of time. By comparison, intravenous administration shows the advantage of a faster onset of action and would be beneficial for rapid clinical control of the condition.

## 4. Conclusion

In the study, an efficient and sensitive analysis method of HPLC-MS/MS was developed and optimized to determine galgravin in plasma after single-dose oral and intravenous administration to rats. The described method had been proved to be successful in studying the bioavailability of galgravin. The absolute bioavailability of 8.5% indicated oral malabsorption of galgravin. This paper provides the first pharmacokinetic and bioavailability study of galgravin, which might be helpful for its further research and applications.

## Figures and Tables

**Table 1:** Mass spectrum parameters of galgravin and IS.

Compound	Parent ion (m/z)	Product ion (m/z)	Collision energy (V)	Fragmentor (V)	Ion mode
Galgravin	373.3	235.2	114	9	Positive
Schisandrin	433.2	384.3	100	15	Positive

**Table 2:** Precision and accuracy of galgravin in rat plasma (*n* = 6).

Spiked conc. (ng/mL)	Intraday			Interday		
	Measured conc. (ng/mL)	RE (%)	RSD (%)	Measured conc. (ng/mL)	RE (%)	RSD (%)
2	2.10 ± 0.16	5.0	7.6	2.12 ± 0.18	6.0	8.5
50	50.25 ± 0.89	0.5	1.8	51.32 ± 3.14	2.6	6.1
400	401.08 ± 15.63	0.3	3.9	389.68 ± 26.24	-2.6	6.7

**Table 3:** Extraction recovery and matrix effects of galgravin in rat plasma (*n* = 6).

Spiked conc. (ng/mL)	Extraction recovery (%)	RSD (%)	Matrix effects (%)	RSD (%)
2	94.4 ± 3.6	3.8	100.0 ± 6.1	6.1
50	99.3 ± 6.5	6.5	96.6 ± 6.3	6.5
400	92.3 ± 5.7	6.2	83.1 ± 5.1	6.1

**Table 4:** Stability data of galgravin in plasma (*n* = 5).

Spiked conc. (ng/mL)	Short-term (2 h)		Freeze-thaw cycles		Autosampler (12 h)		Long-term (7 days)	
	Measured (ng/mL)	RSD (%)	Measured (ng/mL)	RSD (%)	Measured (ng/mL)	RSD (%)	Measured (ng/mL)	RSD (%)
2	1.88 ± 0.22	11.7	2.14 ± 0.20	9.3	1.91 ± 0.10	5.2	2.23 ± 0.27	12.1
50	46.31 ± 4.58	9.9	51.91 ± 2.26	4.4	50.67 ± 3.00	5.9	54.58 ± 3.31	6.1
400	401.15 ± 24.65	6.1	377.38 ± 45.31	12.0	376.18 ± 17.24	4.6	416.77 ± 11.44	2.7

**Table 5:** Pharmacokinetic parameters of galgravin following single oral and intravenous administration to rats (mean ± SD, *n* = 6).

Parameters	Oral administration (20 mg/kg)	Intravenous (2 mg/kg)
AUC_(0 − *t*)_ (h ng/mL)	314.25 ± 179.84	369.56 ± 66.06
AUC_(0 − ∞)_ (h ng/mL)	315.75 ± 179.70	371.36 ± 66.59
MRT_(0 − *t*)_ (h)	7.59 ± 1.67	1.27 ± 0.19
MRT_(0 − ∞)_ (h)	7.80 ± 1.75	1.34 ± 0.21
*T*_1/2*z*_ (h)	3.99 ± 2.08	1.96 ± 0.80
*T*_max_ (h)	2.08 ± 1.56	0.03 ± 0.00
*C*_max_ (ng/mL)	48.42 ± 37.66	332.80 ± 63.59
*F* (%)	8.5	—

AUC: area under the concentration-time curve; MRT: mean residence time; *T*_1/2*z*_: elimination half-life; *C*_max_: maximum concentration; *T*_max_: time of peak concentration; *F*: absolute bioavailability.

## Data Availability

The data used to support the findings of this study are available from the corresponding authors upon request.

## References

[1] Lu Y. Y., Xue Y. B., Chen S. J. (2016). Antioxidant lignans and neolignans from Acorus tatarinowii. *Scientific Reports*.

[2] Majdalawieh A. F., Massri M., Nasrallah G. K. (2017). A comprehensive review on the anti-cancer properties and mechanisms of action of sesamin, a lignan in sesame seeds (Sesamum indicum). *European Journal of Pharmacology*.

[3] Li F. H., Zhang T., Sun H. (2017). A new nortriterpenoid, a sesquiterpene and hepatoprotective lignans isolated from the fruit of Schisandra chinensis. *Molecules*.

[4] da Silva Filho A. A., Costa E. S., Cunha W. R., Silva M. L. A., Nanayakkara N. P. D., Bastos J. K. (2008). In vitro antileishmanial and antimalarial activities of tetrahydrofuran lignans isolated from *Nectandra megapotamica* (Lauraceae). *Phytotherapy Research*.

[5] Zhao Y., Ruan J. L. (2006). Chemical constituents from Piper wallichii. *Journal of Chinese Pharmaceutical Sciences*.

[6] Ikewuchi C. C., Ikewuchi J. C., Ifeanacho M. O. (2015). Phytochemical composition of *Tridax procumbens* Linn leaves: potential as a functional food. *Food and Nutrition Sciences*.

[7] Dong Y., Shi R. F., Liu B. (2007). Study on chemical compositions of Acorus tatarinowii Schott (I). *Journal of Beijing University of Traditional Chinese Medicine*.

[8] Xu L. J., Liu H. T., Peng Y. (2008). Chemical constituents from stems of Schisandra propinqua. *Zhongguo Zhongyao Zazhi*.

[9] Chen Z. N., Yu P. Z., Xu P. J. (1993). Anti-platelet activating factor constituents, 2, 5-diaryltetrahydrofuran type lignans, from Piper futokadsura Sied. et Zucc. *Zhongguo Zhongyao Zazhi*.

[10] da Silva Filho A. A., Andrade e Silva M. L., Carvalho J. C. T., Bastos J. K. (2010). Evaluation of analgesic and anti-inflammatory activities of *Nectandra megapotamica* (Lauraceae) in mice and rats. *The Journal of Pharmacy and Pharmacology*.

[11] Ikewuchi J. C., Ikewuchi C. C., Ifeanacho M. O., Igboh N. M., Ijeh I. I. (2013). Moderation of hematological and plasma biochemical indices of sub-chronic salt-loaded rats by aqueous extract of the sclerotia of Pleurotus tuberregium (Fr) Sing’s: implications for the reduction of cardiovascular risk. *Journal of Ethnopharmacology*.

[12] Zhai H. F., Inoue T., Moriyama M., Esumi T., Mitsumoto Y., Fukuyama Y. (2005). Neuroprotective effects of 2, 5-diaryl-3, 4-dimethyltetrahydrofuran neolignans. *Biological & Pharmaceutical Bulletin*.

[13] Asai M., Lee J. W., Itakura Y. (2012). Effects of veraguensin and galgravin on osteoclast differentiation and function. *Cytotechnology*.

[14] Ponci V., Figueiredo C. R., Massaoka M. H. (2015). Neolignans from Nectandra megapotamica (Lauraceae) display in vitro cytotoxic activity and induce apoptosis in leukemia cells. *Molecules*.

[15] Xin H. X., Dai Z. S., Cai J. F. (2017). Rapid purification of diastereoisomers from Piper kadsura using supercritical fluid chromatography with chiral stationary phases. *Journal of Chromatography A*.

[16] Dong Y., Shi R. B., Liu B. (2008). Determination of the content of galgravin and veraguensin in the effective part of Acorus tatarinowii. *Journal of Beijing University of Chinese Medicine*.

